# Erratum to "In time: averting the legacy of kidney disease - focus on childhood" [Rev. Paul. Pediatr. 34 (1) (2016) 5-10]

**DOI:** 10.1016/j.rppede.2016.04.004

**Published:** 2016

**Authors:** Julie R Ingelfinger, Kamyar Kalantar-Zadeh, Franz Schaefer, World Kidney Day Steering Committee

**Affiliations:** a Massachusetts General Hospital, Boston, USA; b University of California, Irvine, USA; c Heidelberg University Hospital, Heildelberg, Germany

In the editorial **Erratum of "In time: averting the legacy of kidney disease − focus on childhood" [Rev Paul Pediatr. 2016;34(1):5-10]**, in the Table 2, which reads:

**Table 2:** Etiology of chronic kidney disease in children.^2^

DRC			DRET	
Etiology	Percentage (range)		Etiology	Percentage (range)
CAKUT	48-59%		CAKUT	34-43%
GN	5-14%		GN	15-29%
HN	10-19%		HN	12-22%
HUS	2-6%		HUS	2-6%
Cystic	5-9%		Cystic	6-12%
Ischemic	2-4%		Ischemic	2%

CKD, chronic kidney disease; ESRD, childhood onset end-stage renal disease; CAKUT, congenital anomalies of the kidney and urinary tract;GN, glomerulonephritis; HN, hypertension; HUS, hemolytic uremic syndrome. Rare causes include congenital NS, metabolic diseases,cystinosis. Miscellaneous causes depend on how such entities are classified. Chronic Kidney Disease data are from North American Pediatric Renal Trials and Collaborative Studies, the Italian Registry and the BelgianRegistry. Childhood onset end-stage renal disease data are from ANZDATA, ESPN/ERA-EDTA, UK Renal Registry and the Japanese Registry.

It should read:

**Table 2:** Etiology of chronic kidney disease in children.^2^

DRC			DRET	
Etiology	Percentage (range)		Etiology	Percentage (range)
CAKUT	48-59%		CAKUT	34-43%
GN	5-14%		GN	15-29%
NH	10-19%		NH	12-22%
SHU	2-6%		SHU	2-6%
Cystic	5-9%		Cystic	6-12%
Ischemic	2-4%		Ischemic	2%

CKD, chronic kidney disease; ESRD, childhood onset end-stage renal disease; CAKUT, congenital anomalies of the kidney and urinary tract;GN, glomerulonephritis; HN, hereditary nephropathy; HUS, hemolytic uremic syndrome. Rare causes include congenital NS, metabolicdiseases, cystinosis. Miscellaneous causes depend on how such entities are classified. Chronic Kidney Disease data are from North American Pediatric Renal Trials and Collaborative Studies, the Italian Registry and the BelgianRegistry. Childhood onset end-stage renal disease data are from ANZDATA, ESPN/ERA-EDTA, UK Renal Registry and the Japanese Registry.

