# Supplementary material for: In time: averting the legacy of kidney disease – focus on childhood
Source: Rev Paul Pediatr. 2016 Jan-Mar;34(1):5–10. doi: 10.1016/j.rppede.2015.12.001 (PMC4795715; doi:10.1016/j.rppede.2015.12.001)
Supplement: Translation

# Translation: Em tempo: evitando as consequências da doença renal – foco na infância

**Authors:** Julie R. Ingelfinger, Kamyar Kalantar-Zadeh, Franz Schaefer, World Kidney Day Steering Committee

**Affiliations:** a Massachusetts General Hospital, Boston, EUA; b University of California, Irvine, EUA; c Heidelberg University Hospital, Heildelberg, Alemanha

“Pois em cada adulto mora a criança de outrora e em cada criança há o adulto
que ela será”.
John Connolly, O livro das coisas perdidas.

## Introdução e visão geral

O 11° Dia Mundial do Rim será comemorado em 10 de março de 2016, em todo o mundo. Esse evento anual, patrocinado conjuntamente pela Sociedade Internacional de Nefrologia (ISN) e a Federação Internacional de Fundações Renais (IFKF), tornou-se um esforço bem-sucedido para informar ao público em geral e aos legisladores políticos sobre a importância e as ramificações da doença renal. Em 2016, o Dia Mundial do Rim será dedicado à doença renal na infância e os antecedentes da doença renal no adulto, que podem começar na mais tenra infância.

Crianças que sofrem de insuficiência renal aguda (IRA) devido a uma ampla variedade de condições podem ter sequelas em longo prazo que podem levar à doença renal crônica (DRC), muitos anos depois.^1^^-^ 4 Além disso, a DRC na infância, em sua maior parte congênita e complicações de muitas doenças não renais que podem afetar os rins secundariamente, não só levam a significativa morbidade e mortalidade na infância, mas também resultam em problemas médicos que se estendem além da infância. De fato, as mortes na infância de uma longa lista de doenças transmissíveis estão inextricavelmente ligadas ao envolvimento renal. Por exemplo, crianças que sucumbem à cólera e outras infecções diarreicas frequentemente morrem, não por causa da infecção, mas por causa da IRA induzida pela depleção de volume e choque. Além disso, um corpo substancial de dados indica que a hipertensão, proteinúria e DRC na idade adulta têm antecedentes na infância – já a partir da vida fetal e perinatal (ver tabela 1 para definições de infância). O Dia Mundial do Rim em 2016 visa a aumentar a conscientização geral de que muito da doença renal em adultos é, na verdade, iniciada na infância. Compreender os diagnósticos de alto risco e eventos que ocorrem na infância tem o potencial para identificar e intervir preventivamente nas pessoas em maior risco de DRC durante suas vidas.

**Tabela 1:** Definições dos estágios do início da vida

Período perinatal	22 semanas completas de gestação até o 7° dia de vida pós‐natal
Período neonatal	Nascimento até o 28° dia de vida pós‐natal
1^a^ Infância	Nascimento até um ano
Infância	Um ano até 10 anos
Adolescência	10 anos até 19 anos

Obs: Os dados apresentados nessa tabela são mostrados da forma como são definidos pela Organização Mundial de Saúde. Há uma variação em todo o mundo na forma como essas fases do início da vida são definidas. Alguns poderiam definir “jovens indivíduos” como aqueles com 24 anos ou menos. Nos Estados Unidos, a infância como um todo é definida como o período que vai até os 21 anos.

Dados epidemiológicos mundiais sobre o espectro de DRC e IRA em crianças são atualmente limitados, embora seu escopo esteja aumentando. A prevalência de DRC na infância é rara e tem sido relatada como variando de 15-74,7 por milhão de crianças.^3^ Tal variação é provavelmente devida porque os dados sobre DRC são influenciados por fatores regionais e culturais, bem como pela metodologia usada para gerá-los. A Organização Mundial da Saúde (OMS) recentemente adicionou doença renal e urológica à informação sobre mortalidade rastreada em todo o mundo e deve ser uma valiosa fonte de tais dados ao longo do tempo – e mesmo assim a OMS não publica a informação por faixa etária.^5^ Bases de dados, tais como o North American Pediatric Renal Trials and Collaborative Studies (NAPRTCS),^6^ o Sistema de Dados Renais dos Estados Unidos (U.S. Renal Data System – USRDS)^7^ e o registo EDTA^8^ incluem dados sobre doença renal em estágio terminal (DRET) pediátrica e alguns sobre DRC. Projetos como os estudos ItalKid^9^, Chronic Kidney Disease in Children (CKiD)^10^ e Global Burden of Disease Study 2013, bem como os registros que já existem em muitos países, fornecem informações importantes e mais estudos são necessários.^11^

A IRA pode levar à DRC, de acordo com estudos populacionais adultos selecionados^12^ A incidência de IRA em crianças admitidas em unidade de terapia intensiva varia muito – de 8% a 89%^1^ O desfecho depende dos recursos disponíveis. Os resultados de projetos tais como o estudo AWARE, um estudo de cinco nações sobre IRA em crianças, são aguardados.^13^ Estudos unicêntricos, bem como metanálises, indicam que tanto IRA quanto DRC em crianças representam uma minoria de DRC em todo o mundo.^2^^,^ 3 No entanto, é cada vez mais evidente que a doença renal na idade adulta muitas vezes surge de um legado da infância.

## Espectro de doenças renais pediátricas

As condições que respondem por DRC na infância, com predomínio de distúrbios congênitos e hereditários, diferem substancialmente daquelas observadas em adultos. Até o presente, foram descobertas mutações em mais de 150 genes que alteram o desenvolvimento renal ou funções glomerulares ou tubulares específicas.^14^ A maioria desses distúrbios genéticos aparece durante a infância e muitos levam à DRC progressiva. As anomalias congênitas do trato urinário (CAKUT, do inglês *congenital anomalies of kidney and urinary tract*) constituem a maior categoria de DRC em crianças (tabela 2) e incluem hipoplasia/displasia renal e uropatia obstrutiva. Subgrupos importantes entre as displasias renais são as doenças renais císticas, que se originam de defeitos genéticos nos cílios primários das células túbulo-epiteliais. Muitas glomerulopatias pediátricas são causadas por defeitos genéticos ou adquiridos dos podócitos renais, um tipo de célula especial que reveste os capilares glomerulares. Causas menos comuns, mas importantes de DRC na infância são distúrbios metabólicos hereditários, como hiperoxalúria e cistinose e síndrome hemolítico-urêmica (SHU) atípica, uma microangiopatia trombótica relacionada a anormalidades genéticas de complemento, coagulação ou vias metabólicas.

**Tabela 2:** Etiologia da doença renal crônica em crianças 2

DRC			DRET	
Etiologia	Porcentagem (Variação)		Etiologia	Porcentagem (Variação)
CAKUT	48‐59%		CAKUT	34‐43%
GN	5‐14%		GN	15‐29%
HN	10‐19%		HN	12‐22%
SHU	2‐6%		HUS	2‐6%
Cística	5‐9%		Cística	6‐12%
Isquêmica	2‐4%		Isquêmica	2%

DRC, doença renal crônica; DRET, doença renal em estágio terminal com início na infância; CAKUT, anomalias congênitas do rim e do trato urinário; GN, glomerulonefrite; HN, hipertensão; SHU, síndrome hemolítico‐urêmica. Causas raras incluem causas congênitas (SN), doenças metabólicas, cistinose. Causas diversas dependem de como tais entidades são classificadas.Dados sobre Doença Renal Crônica foram obtidos dos estudos North American Pediatric Renal Trials and Collaborative Studies, o registro italiano e o registro belga. Dados de doença renal em estágio terminal com início na infância foram obtidos dos estudos ANZDATA, ESPN/ERA‐EDTA, Registro Renal do Reino Unido e o registro Japonês.

Várias classificações não deixam claro como categorizar as crianças que sofreram IRA e aparentemente se recuperaram ou como e se devemos incluir as crianças que tiveram desafios perinatais, provavelmente resulta em um número relativamente baixo de néfrons.

Entre as crianças com doença renal em estágio terminal (DRET) de início na infância, as glomerulopatias são um pouco mais e anomalias congênitas um pouco menos comuns (tabela 2), devido à perda de néfrons tipicamente mais rápida na doença glomerular. No entanto, evidências recentes sugerem que muitos pacientes com formas mais leves de CAKUT podem evoluir para DRET na idade adulta, com pico na quarta década de vida.^15^

Existem diferenças nacionais e regionais nos tipos e curso de IRA e DRC durante a infância e além. Morte por doença renal é maior nos países em desenvolvimento e as disparidades nacionais e regionais no tratamento e os desfechos devem ser abordados. Além disso, o acesso ao tratamento é variável, depende da região, do país e sua infraestrutura. Com a concentração em doença renal na infância, soluções de baixo custo podem ser atingidas, já que o tratamento precoce e preventivo da doença pode impedir mais tarde a DRC mais avançada. As expectativas dependem da disponibilidade de tratamento e gestão. Tratar crianças, mesmo desde a infância, que têm IRA e DRC que requer terapia de substituição renal (TSR) pode ser eficaz na redução do impacto da doença renal na fase adulta. Fazer isso requer recursos que se concentram em maneiras mais rápidas e custo-efetivas de fornecer TSR aguda na infância.

## Doença renal congênita e origens desenvolvimentistas da saúde e doença, dotação renal e implicações

Em regiões onde as ultrassonografias fetais pré-natais são rotineiras, muitas crianças com anormalidades urológicas são identificadas no exame pré-natal, o que permite a intervenção precoce. No entanto, em grande parte do mundo as crianças com anormalidades estruturais não são identificadas até muito mais tarde, quando os sintomas se desenvolvem. Enquanto o rastreio generalizado para proteinúria, hematúria e infecções do trato urinário é feito em alguns países e regiões, há uma falta de consenso quanto à sua eficácia. No entanto, há um consenso de que as crianças com estudos de ultrassom pré-natal que indiquem eventuais anomalias geniturinárias, crianças com histórico familiar de doença renal e crianças com sinais tais como falha do crescimento ou histórico de infecção urinária, disfunção miccional ou urina com aparência anormal devem ser examinadas. O rastreio inicial incluiria um exame físico dedicado e um exame de urina, urina tipo 1 e um painel químico básico, seguido por uma avaliação mais focada, se indicado.

A depender do diagnóstico, terapias definitivas podem ser indicadas. No entanto, a evidência de que a terapia irá retardar a progressão da DRC na infância continua a ser limitada. Inibidores da enzima de conversão da angiotensina, bloqueadores do receptor da angiotensina, antioxidantes e, eventualmente, mudanças na dieta podem ser indicadas, dependem do diagnóstico. Entretanto, mudanças na dieta precisam permitir o crescimento e desenvolvimento adequados. O estudo ESCAPE forneceu evidências de que o controle rigoroso da pressão arterial retarda a progressão da DRC em crianças, independentemente do tipo de doença renal subjacente.^16^

Algumas crianças muito pequenas podem necessitar de TSR na primeira infância. Dados recentes compilados a partir de registros em todo o mundo indicam boa sobrevida, mesmo quando a diálise é necessária desde a fase neonatal.^2^^,^ 17 O transplante renal, a terapia de substituição renal preferida em crianças, é geralmente adequado após 12 meses de idade, com excelente sobrevida do paciente e do enxerto, crescimento e desenvolvimento (fig. 1).

**Figura 1:** Os tipos e riscos da doença renal mudam durante o ciclo da vida. A contribuição do número de néfrons aumenta ao longo do ciclo da vida, em conjunto com eventos que causam insultos diretos e desafios para a saúde renal.

Há cada vez mais evidências de que a DRC com início na infância leva à morbidade cardiovascular acelerada e expectativa de vida mais curta. Espera-se que grandes estudos prospectivos em curso, tais como o Cardiovascular Comorbidity in Children with CKD (4C), informem sobre as causas e consequências da doença cardiovascular precoce em crianças com DRC.^18^

Além dessas crianças com doença renal congênita, sabe-se agora que os eventos perinatais podem afetar a saúde futura, na ausência de doença renal evidente no início da vida.^19^ Os bebês prematuros parecem estar particularmente em risco para doença renal muito tempo depois do nascimento, com base tanto em estudos de coorte observacionais, bem como relatos de casos. Cada vez mais prematuros sobrevivem, entre eles muitos nascidos bem antes de a nefrogênese estar completa.^20^ Os dados disponíveis são limitados e indicam que no processo de tratamento na UTI neonatal esses bebês recebem muitas nefrotoxinas e que aqueles que morrem antes da alta têm menos e maiores glomérulos.^21^ Além disso, os sobreviventes têm evidência de insuficiência renal que pode ser sutil.^22^ Ainda mais preocupante, muitos dados epidemiológicos indicam que pessoas nascidas a termo, mas com peso ao nascer relativamente baixo, podem ser de alto risco para hipertensão, albuminúria e DRC em fase posterior da vida.^23^ Quando medidas diretas são tomadas, tais pessoas, quando adultas, podem ter menos néfrons e, portanto, uma baixa dotação cardiorrenal.

Ao concentrar-se nas crianças no Dia Mundial do Rim, gostaríamos de observar que essa é a chave para avaliar a função renal e pressão arterial ao longo da vida dessas pessoas nascidas prematuramente ou pequenas para a idade gestacional. Ao fazê-lo, e evitando medicamentos nefrotóxicos ao longo da vida, pode ser possível evitar a DRC em muitas pessoas.

## Recursos e terapêutica para crianças – Diferenças da terapêutica em adultos

Existem disparidades na disponibilidade de recursos para tratar IRA em crianças e jovens; consequentemente, muitas crianças e adultos jovens nos países em desenvolvimento morrem quando IRA ocorre. Para resolver o problema, o ISN iniciou o Saving Young Lives Project (Projeto Salvando Jovens Vidas), que visa tanto a prevenir a IRA com o tratamento imediato de infecção e/ou administração apropriada de terapia de fluidos e eletrólitos como tratar a IRA quando ela ocorre. Esse projeto em andamento na África Subsaariana e no Sudeste Asiático, do qual quatro fundações renais participam igualmente [IPNA (International Pediatric Nephrology Association), ISN (International Society of Nephrology), ISPD (International Society for Peritoneal Dialysis), SKCF (Sustainable Kidney Care Foundation)], centra-se na criação e manutenção de centros para o tratamento da IRA, incluindo o fornecimento de diálise peritoneal aguda. Ele está associado com o projeto do ISN 0 em 25, que convida os membros a assegurarem que em 2025 ninguém mais morra por insuficiência renal aguda e evitável.

Considerando a preponderância de distúrbios congênitos e hereditários, os recursos terapêuticos para crianças com DRC historicamente têm sido limitados a algumas condições imunológicas. Muito recentemente, o progresso no desenvolvimento de medicamentos em conjunto com avanços no conhecimento genético e capacidade de diagnóstico começou a superar o longo “niilismo terapêutico” na doença renal pediátrica. A SHU atípica, considerada por muito tempo como uma condição fatídica, com alta probabilidade de progressão para doença renal de estágio terminal e recorrência pós-transplante, transformou-se em uma condição tratável – com o advento de um anticorpo monoclonal que bloqueia especificamente a ativação de C5.^24^ Outro exemplo é o uso de. antagonistas do receptor da vasopressina para retardar o crescimento do cisto e preservar a função renal na doença renal policística.^25^ Primeiro a ser demonstrado como eficaz em adultos com doença renal policística autossômica dominante, o tratamento com vaptanos parece ser promissor também para a forma recessiva da doença, que se apresenta e muitas vezes progride para doença renal de estágio terminal durante a infância.

No entanto, o benefício para os pacientes resultantes das descobertas das pesquisas farmacológicas é comprometido em uma escala global pelo enorme custo de alguns dos novos agentes terapêuticos. A busca de terapias inovadoras a preços acessíveis para as doenças raras será uma questão chave em nefrologia pediátrica nos próximos anos.

A identificação de crianças que possam se beneficiar das novas abordagens terapêuticas será muito facilitada pelo desenvolvimento de registros clínicos que informem sobre o curso natural da doença, incluindo correlações genótipo-fenótipo. Além dos bancos de dados de doenças específicas, há também uma necessidade de registros específicos para o tratamento. Esses são particularmente relevantes em áreas onde os estudos clínicos são difíceis de executar devido ao número pequeno de pacientes e à falta de interesse da indústria, bem como para as terapias que necessitam de desenvolvimento ou melhoria global. Por exemplo, há atualmente grande gradiente internacional na penetração e desempenho da diálise e transplante pediátricos. Considerando que as taxas de sobrevivência dos pacientes e as técnicas pediátricas são excelentes e até mesmo superiores às dos adultos em muitos países industrializados, estima-se que quase metade da população infantil do mundo não recebe terapia de substituição renal crônica em absoluto. O fornecimento de acesso à TSR para todas as crianças será um enorme desafio no futuro. Para obter informações confiáveis sobre a demografia e os desfechos de TSR em pacientes pediátricos, a International Pediatric Nephrology Association (IPNA) está prestes a lançar um registro de base populacional global. Se for bem-sucedido, o registro da IPNA sobre TSR pode se tornar um modelo para coleta de dados global.

## Transição do tratamento pediátrico para o tratamento na vida adulta

A transição do tratamento de adolescentes com doença renal para um cenário adulto é fundamental tanto para os pacientes quanto para seus cuidadores. A não adesão é um marco muito frequente de transição do tratamento pediátrico para atendimento de adultos em pacientes jovens com doenças crônicas.^26^^-^ 28 Assim, medidas ponderadas, combinadas com procedimentos definidos sistematicamente e apoiados por vias validadas e diretrizes confiáveis devem ser instituídos para garantir resultados bem-sucedidos.

O processo de mudança de tratamento pediátrico para a “transição” para o tratamento de adultos, que deverá ocorrer gradualmente, deve ser distinguido da “transferência”, que é muitas vezes uma mudança abrupta e mecanicista no cenário do provedor. Introduzir o conceito de transição deve ser a preferência, iniciando-se meses e até anos antes do tempo alvo, à medida que as crianças passam pela adolescência e a idade adulta. O objetivo final é promover um relacionamento forte e um plano individualizado no novo ambiente que permita que o paciente se sinta confortável o suficiente para relatar a não adesão e outros lapsos de tratamento.

Um plano de transição deve reconhecer que a maturidade emocional das crianças com doença renal pode diferir de forma ampla. A avaliação do cuidador e da estrutura familiar, bem como fatores culturais, sociais e financeiros no momento da transição, é fundamental, inclui uma avaliação realista do impacto para o cuidador.^4^ O tempo e formato adequados da transição podem variar amplamente entre os diferentes pacientes e em diferentes contextos; assim, um processo flexível sem uma data definida e mesmo sem um formato delineado pode ser preferível.

É importante ressaltar que a transição pode necessitar ser feita de forma mais vagarosa, parar ou até mesmo ser revertida temporariamente durante as crises, tais como exacerbações ou progressão da doença, ou se ocorrerem instabilidades familiares ou sociais. A recente declaração conjunta de consenso da International Society of Nephrology (ISN) e International Pediatric Nephrology Association (IPNA) têm proposto medidas coerentes com os pontos esboçados, com o objetivo de melhorar a transição dos cuidados em doença renal na prática clínica.^29^^,^ 30

## Convite para a geração de mais informações e ações

Considerando as vulnerabilidades das crianças com doença renal, incluindo o impacto no crescimento e desenvolvimento e a vida futura como indivíduo adulto, e dada a proporção muito maior de crianças nos países em desenvolvimento que enfrentam limitações de recursos, a educação de todos os envolvidos é imprescindível, a fim de realinhar as comunicações e ações.^31^^,^ 32 Esses esforços devem promover colaborações regionais e internacionais e intercâmbio de ideias entre fundações renais locais, sociedades profissionais, organizações sem fins lucrativos e estados e governos, de modo a ajudar a capacitar todas as partes interessadas para melhorar a saúde, o bem-estar e qualidade de vida de crianças com doenças renais e para garantir a sua longevidade na vida adulta.

Até recentemente, porém, a declaração de consenso da OMS sobre doenças não transmissíveis (DNT) incluiu doenças cardiovasculares, câncer, diabete e doença respiratória crônica, mas não doença renal.^33^^,^ 34 Felizmente, devido, em parte, a uma campanha global liderada pelo ISN, a Declaração Política sobre DNT da Cúpula das Nações Unidas em 2011 mencionou a doença renal no Item 19.^35^

Aumentar a educação e conscientização sobre doenças renais na doença geral e renal na infância, em particular, é consistente com os objetivos da OMS para reduzir a mortalidade por DNT com iniciativas em nível da população-alvo de 10 anos com foco em mudanças no estilo de vida (incluindo a redução do consumo de tabaco, controle de consumo de sal, controle de energia da dieta e redução de ingestão de álcool) e intervenções efetivas (incluindo pressão arterial, colesterol e controle glicêmico).

São necessários esforços aumentados para realinhar e expandir essas colaborações multidisciplinares, com foco mais efetivo na detecção e no tratamento precoces da doença renal em crianças no início. Considerando que as questões relacionadas à doença renal podem ser ofuscadas por outras DNT com aparentemente maiores implicações na saúde pública, tais como diabete, câncer e doenças cardiovasculares, nossos esforços também devem aumentar a educação e sensibilização em tais condições que se sobrepõem, como as associações cardiorrenais, a natureza global da DRC e DRET como as principais doenças não transmissíveis, bem como o papel da insuficiência renal como doença multiplicadora e confundidora de outras doenças não transmissíveis. Publicações governamentais, incluindo artigos de consenso e revisões de diagramas de especialistas de classe mundial, podem servir para aprimorar essas metas.^36^
